# Yeast screening system reveals the inhibitory mechanism of cancer cell proliferation by benzyl isothiocyanate through down-regulation of Mis12

**DOI:** 10.1038/s41598-019-45248-2

**Published:** 2019-06-20

**Authors:** Naomi Abe-Kanoh, Narumi Kunisue, Takumi Myojin, Ayako Chino, Shintaro Munemasa, Yoshiyuki Murata, Ayano Satoh, Hisao Moriya, Yoshimasa Nakamura

**Affiliations:** 1 Graduate School of Environmental and Life Science, Okayama University, Okayama, 700-8530 Japan; 2 Research Fellow of Japan Society for the Promotion of Science, Chiyoda-ku, Tokyo, 102-0083 Japan; 3 Institute of Biomedical Sciences, Tokushima University Graduate School, Tokushima, 770-8503 Japan; 4 Research Core for Interdisciplinary Sciences, Okayama University, Okayama, 700-8530 Japan; 5 Graduate School of Natural Science and Technology, Okayama University, Okayama, 700-8530 Japan

**Keywords:** Mechanism of action, Target identification, Molecular medicine

## Abstract

Benzyl isothiocyanate (BITC) is a naturally-occurring isothiocyanate derived from cruciferous vegetables. BITC has been reported to inhibit the proliferation of various cancer cells, which is believed to be important for the inhibition of tumorigenesis. However, the detailed mechanisms of action remain unclear. In this study, we employed a budding yeast *Saccharomyces cerevisiae* as a model organism for screening. Twelve genes including *MTW1* were identified as the overexpression suppressors for the antiproliferative effect of BITC using the genome-wide multi-copy plasmid collection for *S. cerevisiae*. Overexpression of the kinetochore protein Mtw1 counteracts the antiproliferative effect of BITC in yeast. The inhibitory effect of BITC on the proliferation of human colon cancer HCT-116 cells was consistently suppressed by the overexpression of Mis12, a human orthologue of Mtw1, and enhanced by the knockdown of Mis12. We also found that BITC increased the phosphorylated and ubiquitinated Mis12 level with consequent reduction of Mis12, suggesting that BITC degrades Mis12 through an ubiquitin-proteasome system. Furthermore, cell cycle analysis showed that the change in the Mis12 level affected the cell cycle distribution and the sensitivity to the BITC-induced apoptosis. These results provide evidence that BITC suppresses cell proliferation through the post-transcriptional regulation of the kinetochore protein Mis12.

## Introduction

Naturally-occurring isothiocyanates (ITCs), such as benzyl ITC (BITC), phenethyl ITC (PEITC) and sulforaphane (SFN) derived from cruciferous vegetables, have been demonstrated to block the tumor formation initiated by chemicals in experimental animals, and the dietary consumption of ITCs has been shown to strongly correlate with the reduced risk of various cancers in humans^1–4^. The antiproliferative effect of ITCs through the induction of cell cycle arrest and apoptosis is believed to be one of the important determinants for the inhibition of tumorigenesis. For example, BITC induces cell cycle arrest at the G_2_/M phase by a p38 mitogen-activated protein kinase (MAPK)-dependent pathway in human leukemia Jurkat cells^5^ and by an extracellular signaling-regulated kinase-dependent pathway in human pancreatic cancer Capan-2 cells^6^. Also BITC induces apoptosis by the mitochondrial death pathway in rat liver epithelial RL34 cells^7^ and by a c-Jun-*N*-terminal kinase-dependent pathway in Jurkat cells. However, the detailed molecular mechanism involved in the antiproliferative effect of ITCs remains to be clarified.

The identification of molecular targets of ITCs conferring their antiproliferative ability contributes to the understanding of the molecular basis of ITCs^8^. As electrophiles, ITCs can covalently bind to cellular nucleophilic amino acids, such as Cys and Lys. Mi *et al*. found a correlation between the covalent binding to the cellular proteins and antiproliferative effect of ITCs^9^. Consistent with this report, the direct binding of ITCs to several proteins, such as tubulin^10^, macrophage migration inhibitory factor^11^ and DNA topoisomerase II alpha^12^, are likely to be associated with the antiproliferative ability of ITCs. Proteomics analysis by 2-dimensional gel electrophoresis has recently been used to screen the binding targets of ITCs and identified 30 proteins as the binding targets of ITCs *in vitro*^13^. However, the binding targets of ITCs identified by proteomics include several proteins abundantly existing in the cells, such as actin, tubulin and vimentin^14^. The low binding specificity of ITCs makes it difficult to find the molecular targets specifically contributing to the antiproliferative effect of ITCs. Therefore, the paradigm shift of the screening system is needed in terms of the requirement of the targets for the ITC-induced phenotype.

Budding yeast *Saccharomyces cerevisiae* is a eukaryotic model organism which has been frequently used in scientific studies due to its easy and cheap cultivation, short generation time and the ease of the application of molecular techniques for its genetic manipulation^15,16^, and widely employed for the identification of drug targets and mechanism of action studies^17^. The yeast screening system would be especially useful for the identification of target molecules contributing to the antiproliferation by ITCs, because ITCs exert an antiproliferative effect in yeast as well as in human cancer cells^18^, and antiproliferative agents often target the components of cell division and DNA repair machineries which are highly conserved between humans and yeast. One of the approaches to identify small-molecule targets is a multi-copy suppression screening for genes that provide resistance to a drug on overexpression. This screening is based on the principle that cells overexpressing a small-molecule target should tolerate the higher levels of the drug^19^. In addition, the yeast genome has been entirely sequenced and includes about 6000 open reading frames (ORFs)^20,21^. Based on the genome, we previously developed pRS423ks, a genome-wide multi-copy plasmid collection of *S. cerevisiae*^22^. Briefly, each gene with its native regulatory elements (promoter and terminator) is cloned to a 2-micron-based plasmid vector. Plasmid vectors derived from a 2-micron circle, which is a naturally-observed selfish DNA in *S. cerevisiae*, have copy numbers of 10 to 40 per cell^23^. Although the promoter swapping using the strong promoter, such as *GAL1*, causes an absolute gene overexpression, pRS423ks causes a relative gene overexpression from the native level. We then tried to identify the molecular targets for the antiproliferative effect of BITC by the introduction of the plasmid collection to the wild-type yeast strain and the following examination of individual transformants for resistance to BITC.

To investigate the mechanisms of antiproliferation by BITC in human cancer cells, we conducted a genome-wide overexpression screening in yeast and applied the results obtained from the yeast to human cancer cells. By the screening, 12 genes, including *MTW1* encoding an essential component of the MIND kinetochore complex, were identified as overexpression suppressors of antiproliferation by BITC in yeast. We found that the down-regulation of Mis12, a human orthologue of Mtw1, plays an important role in the antiproliferation by BITC in human colon cancer HCT-116 cells. Our data indicated that the proteasome-dependent decrease in Mis12 induces G_2_/M delay and enhances the BITC-induced apoptosis, which contributes to the suppression of cancer cell proliferation by BITC.

## Results

### BITC dose-dependently suppresses yeast cell growth

**Figure 1:**
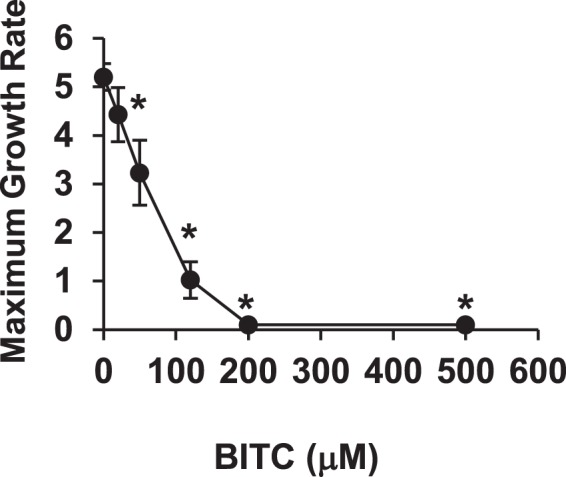
BITC inhibits cell growth in yeast. Yeast BY4741 cells were incubated in the YPD medium with different concentrations of BITC in a 96 well-plate. The time-lapse change in absorbance at 595 nm was measured using a microplate reader. Based on these data, the maximum growth rate was calculated. The values represent means ± SEM of three separate experiments (*P < 0.05 compared with control; Student’s *t*-test).

To determine the concentration of BITC for the yeast screening, we examined the effect of BITC on the yeast cell growth by calculating the maximum growth rate in the yeast BY4741 strain. As shown in Fig. 1, the maximum growth rate decreased with the increasing concentrations of BITC, which suggests that BITC dose-dependently suppresses the proliferation of yeast. Since the treatment of BITC at a too low or too high concentration makes it difficult to detect the recovery of the maximum growth rate by overexpressing genes, we decided to use 100 μM BITC for the screening.

### Overexpression of 12 genes contributes to BITC resistance in yeast

**Figure 2:**
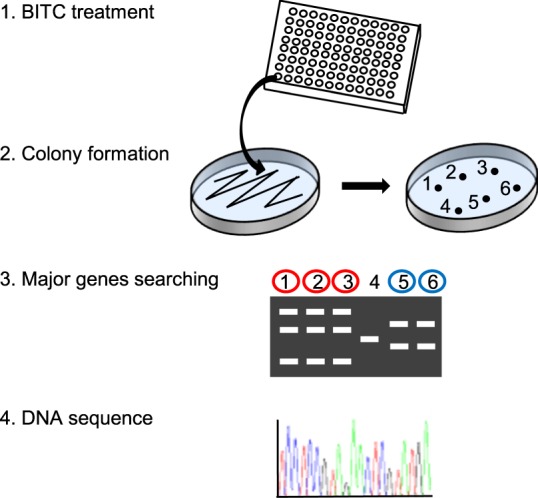
Scheme of screening for overexpression suppressors of antiproliferation by BITC in yeast. Transformants of BY4741 cells with pRS423ks were treated with 100 μM BITC for 3 days in YPD medium. Yeasts from each well were cultured on an SC-His agar plate. Formed colonies were subjected to colony PCR to amplify the inserted DNA into the plasmid. The PCR products were digested with restriction enzymes and separated by gel electrophoresis. Only the plasmid DNA of the samples with a major band fragmentation pattern was subjected to DNA sequencing.

**Figure 3:**
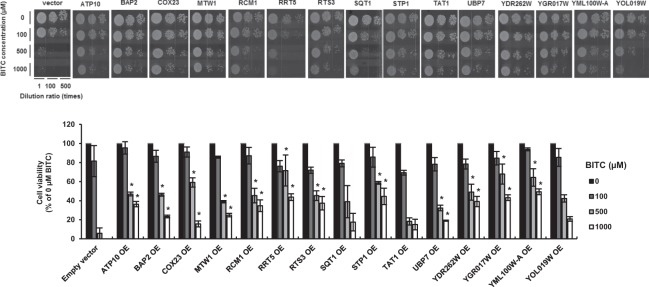
Validation of screening by spot assay. BY4741 cells were transformed with pRS423ks with ORF of each of the 15 genes identified by screening and empty vector (control). The transformants were treated with the indicated concentrations of BITC for 30 min in YPD medium. The cell suspensions were spotted on a YPD agar plate. The number of colonies was counted to determine the cell viability. The values represent means ± SEM of three separate experiments (*P < 0.05 compared between overexpression (OE) group of each gene and control at the same concentrations of BITC; Student’s *t*-test).

The scheme of yeast screening by BITC is shown in Fig. 2. First is a BITC treatment step: transformants of BY4741 cells with 8 groups of pRS423ks, a genome-wide multi-copy plasmid collection of yeast^22^ (about 750 ORFs/group), were treated with 100 μM BITC at 30 °C for 3 days in YPD liquid medium. Second is a colony formation step; a cell suspension from each well was spotted and cultivated on synthetic complete without the His (SC-His) agar plate at 30 °C for 3 days. Third is a major gene searching step; inserted DNA in the plasmid was amplified by colony PCR, digested by restriction enzyme and separated by gel electrophoresis to find the same patterns of the inserted DNA. The plasmid DNA of a sample with the same frequently seen (more than 2 times) band patterns was selected and isolated. Fourth is a DNA sequencing step; 15 gene candidates were identified by DNA sequencing using the isolated plasmids and primers. To validate the screening, pRS423ks plasmids with the sequences of the 15 identified genes were cloned by *Escherichia coli* and introduced to yeast again, then the transformants were subjected to a spot assay. As shown in Fig. 3, overexpression of the 12 genes (*ATP10, BAP2, COX23, MTW1, RCM1, RRT5, RTS3, STP1, UBP7, YDR262W, YGR017W and YML100W-A*), but not overexpression of 3 genes (*SQT1, TAT1 and YOL019W*), significantly weakened the antiproliferative effect of BITC compared to the control group (empty vector), suggesting that the overexpression of these 12 genes contributes to the BITC resistance in yeast (Table 1).

**Table 1:** Overexpression suppresser genes of antiproliferation by BITC in yeast.

Gene	Brief description*
*ATP10*	Assembly factor for mitochondrial F1F0 ATP synthase
*BAP2*	Branched-chain amino acid permease
*COX23*	Copper chaperone for cytochrome c oxidase
*MTW1*	Essential component of the MIND kinetochore complex
*RCM1*	rRNA m5C methyltransferase
*RRT5*	Unknown
*RTS3*	Putative component of protein phosphatase 2A complex
*STP1*	Transcriptional regulator of amino acid transporter genes
*UBP7*	Ubiquitin-specific protease
*YDR262W*	Unknown
*YGR017W*	Unknown
*YML100W-A*	Unknown

^*^Saccharomyces genome database: http://www.yeastgenome.org.

### Change in Mis12 level affects the sensitivity to BITC in human cancer cells

**Figure 4:**
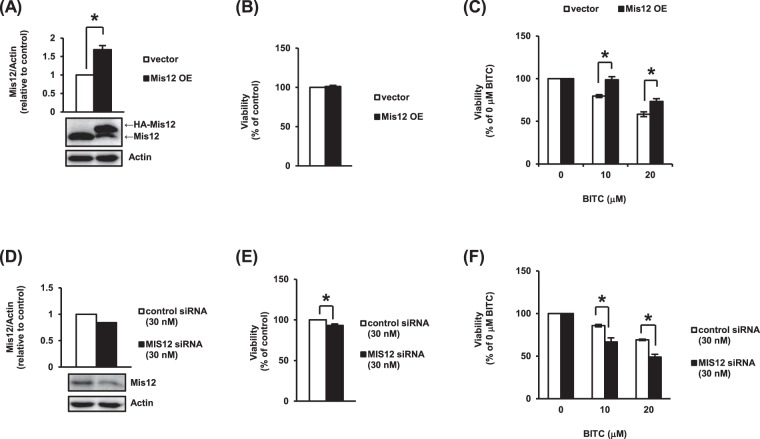
Change in Mis12 protein level affects the sensitivity of cells to the antiproliferative effect of BITC. The Mis12 protein level was determined by a Western blot analysis. Actin was used as a loading control. The viability was determined by a trypan blue dye exclusion assay. (**A**) Mis12 protein level in HCT-116 cells stably expressing HA-Mis12 (Mis12 OE cells) was determined. pQCXIP (vector) was used as the control. (**B**) Mis12 OE and control cells (2 × 10^6^) were cultured for 24 h and the viability was determined. (**C**) Mis12 OE and control cells (2 × 10^6^) were treated with BITC for 24 h and the cell viability was determined. (D&E) HCT-116 cells were transfected with the control siRNA or Mis12-specific siRNA. The Mis12 protein level (**D**) and viability (**E**) were then determined. (**F**) HCT-116 cells were transfected with the control siRNA or Mis12-specific siRNA. After the treatment with BITC for 24 h, the cell viability was determined. The values represent means ± SEM of three separate experiments (*P < 0.05 compared between the indicated groups; Student’s *t*-test).

We focused on *MTW1* among the 12 identified genes because the function and structure of yeast Mtw1 are highly conserved in the human orthologue of Mtw1, Mis12. Mis12, an essential component of the Mis12 kinetochore complex in humans, is required for the appropriate chromosome segregation during mitosis^24^. In human colon cancer HCT-116 cells, we examined the effects of the overexpression and knockdown of Mis12 on the antiproliferation by BITC. The Mis12 protein level in HCT-116 cells stably overexpressing Mis12 (Mis12 OE cells) was about 1.7 times higher than that in the vector control (Fig. 4A). The Mis12 overexpression itself didn’t affect the cell proliferation (Fig. 4B). As shown in Fig. 4C, the antiproliferative effect of BITC in Mis12 OE cells was significantly attenuated compared to the vector control, which is consistent with the result from the yeast in Fig. 3. The transfection of HCT-116 cells with 30 nM Mis12-specific siRNA depleted the Mis12 protein level by 16% compared to control (Fig. 4D). Mis12 knockdown alone weakly, but significantly, suppressed the cell proliferation (Fig. 4E). As shown in Fig. 4F, BITC itself dose-dependently suppressed cell proliferation in the control siRNA-treated group, whereas the Mis12 knockdown enhanced the antiproliferative effect of BITC. These results suggested that the expression level of Mis12 in human as well as Mtw1 in yeast affects the antiproliferative effect of BITC.

### BITC induces proteasome-dependent decrease of Mis12 in HCT-116 cells

**Figure 5:**
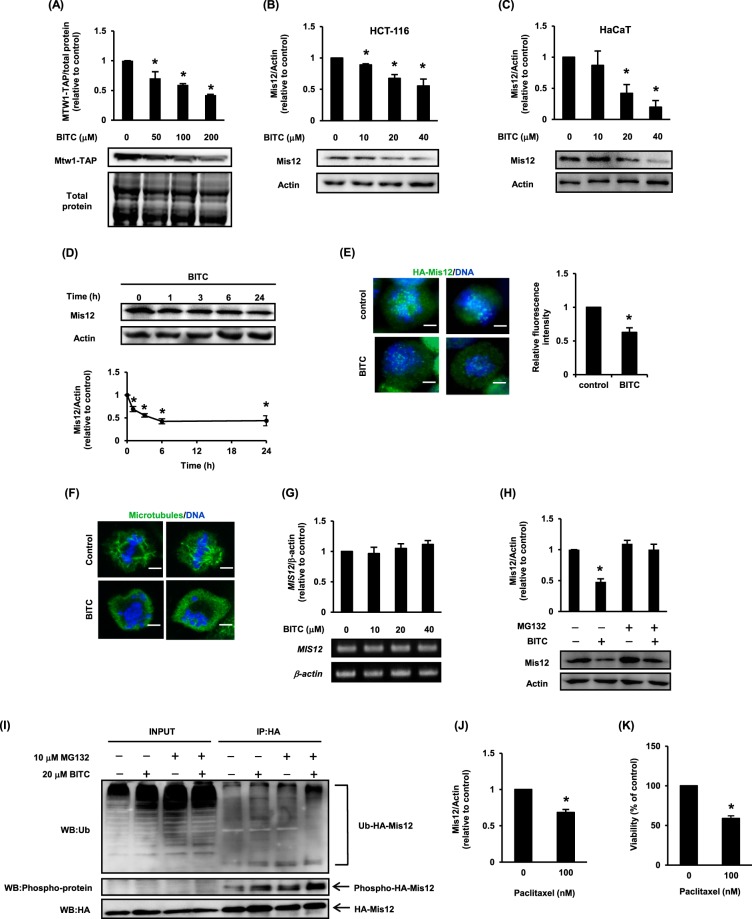
BITC decreases Mis12 protein expression in a proteasome-dependent manner. The Mtw1/Mis12 protein level was determined by a Western blot analysis. Total protein staining or actin was used as the loading control. (**A**) BY4741 cells expressing the Mtw1-TAP were treated with BITC for 24 h and Mtw1-TAP protein level was determined. (**B**) HCT-116 cells were treated with BITC for 24 h and the Mis12 protein level was determined. (**C**) HaCaT cells were treated with BITC for 24 h and the Mis12 protein level was determined. (**D**) HCT-116 cells were treated with 20 μM BITC for the indicated hours and the Mis12 protein level was determined. (**E**) HCT-116 cells stably expressing HA-Mis12 were treated with 20 μM BITC for 1 h. The signals of HA-Mis12 (green) and DNA (blue) were detected by immunofluorescence. The signal intensity of HA-Mis12 at each kinetochore was measured relative to an adjacent background signal. Bar, 10 µm. Fluorescence intensity was quantified using Image J software. (**F**) HCT-116 cells were treated with 20 μM BITC for 1 h. The signals of the microtubules (green) and DNA (blue) were detected by immunofluorescence. Bar, 10 µm. (**G**) HCT-116 cells were treated with BITC for 24 h and the Mis12 mRNA level was determined by RT-PCR. (**H**) HCT-116 cells were treated with 10 μM MG132 and/or 20 μM BITC for 24 h, and the Mis12 protein level was determined. (**I**) HCT-116 cells stably expressing HA-Mis12 were treated with 10 μM MG132 and/or 20 μM BITC for 30 min. The whole cell lysates were immunoprecipitated with the HA antibody and visualized by a Western blot analysis with ubiquitin, phosphoserine/threonine/tyrosine or HA-tag antibody. (**J**) HCT-116 cells were treated with 100 nM paclitaxel for 24 h and subjected to Western blot analysis to detect Mis12 signal. Actin was used as a loading control. (**K**) HCT-116 cells were treated with 100 nM paclitaxel for 24 h and subjected to MTT assay to measure cell viability. The values represent means ± SEM of three separate experiments (*P < 0.05 compared with control; Student’s *t*-test).

We examined the effect of BITC on the protein expression of Mtw1 in yeast and Mis12 in HCT-116 cells. For detection of the Mtw1 protein expression, we used the yeast strain whose *MTW1* sequence on the genome is replaced with the *MTW1-TAP* sequence by homogeneous recombination. Western blot analysis using the anti-TAP-tag and anti-Mis12 antibodies showed that BITC significantly decreased the Mtw1-TAP level in yeast (Fig. 5A) and the Mis12 level not only in HCT-116 cells, but also in immortalized non-tumorigenic human epidermal (HaCaT) cells (Fig. 5B,C). In addition, the anti-cancer agent paclitaxel decreased the Mis12 level and cell viability in HCT-116 cells (Fig. 5J,K). The decrease of Mis12 was observed 1 h after the BITC treatment and continued for 24 h after treatment (Fig. 5D). A previous study indicated that Mis12 appears to localize on DNA throughout the cell cycle^24^. Immunofluorescence data showed that BITC didn’t affect the subcellular localization of Mis12 on DNA but significantly decreased the Mis12 fluorescent signal (Fig. 5E). Although the results in mitotic phase were shown as the representative pictures in Fig. 5E, we also confirmed that the fluorescent foci of Mis12 were observed on DNA in interphase (data not shown). Furthermore, we observed the misaligned chromosome in the BITC-treated HCT-116 cells (Fig. 5F). This finding is consistent with a previous report showing that the siRNA-mediated down-regulation of Mis12 induces misalignment of the chromosomes in HeLa cells^25^.

Next, to check whether or not the decrease of the Mis12 protein level by BITC is regulated by the transcriptional level, we investigated the effect of BITC on the mRNA expression of *MIS12*. As shown in Fig. 5G, BITC showed no significant effect on the *MIS12* mRNA expression in HCT-116 cells. We next examined the effect of proteasome inhibitor, MG132, on the BITC-decreased Mis12 protein level. The co-incubation with MG132 impaired the BITC-decreased expression of the Mis12 protein (Fig. 5H). Since some phosphorylated proteins could be recognized as the substrates of the E3 ubiquitin ligase and degraded through an ubiquitin-proteasome pathway, we performed an immunoprecipitation assay to examine the post-transcriptional modification of Mis12. As expected, BITC drastically increased the phosphorylated Mis12 and tended to induce the ubiquitination of Mis12 (Fig. 5I). These results suggested that BITC degrades the Mis12 protein, possibly through the ubiquitin-proteasome pathway without affecting its gene expression or subcellular localization.

### Down-regulation of Mis12 increases G2/M cell population and enhances BITC-induced apoptosis in HCT-116 cells

**Figure 6:**
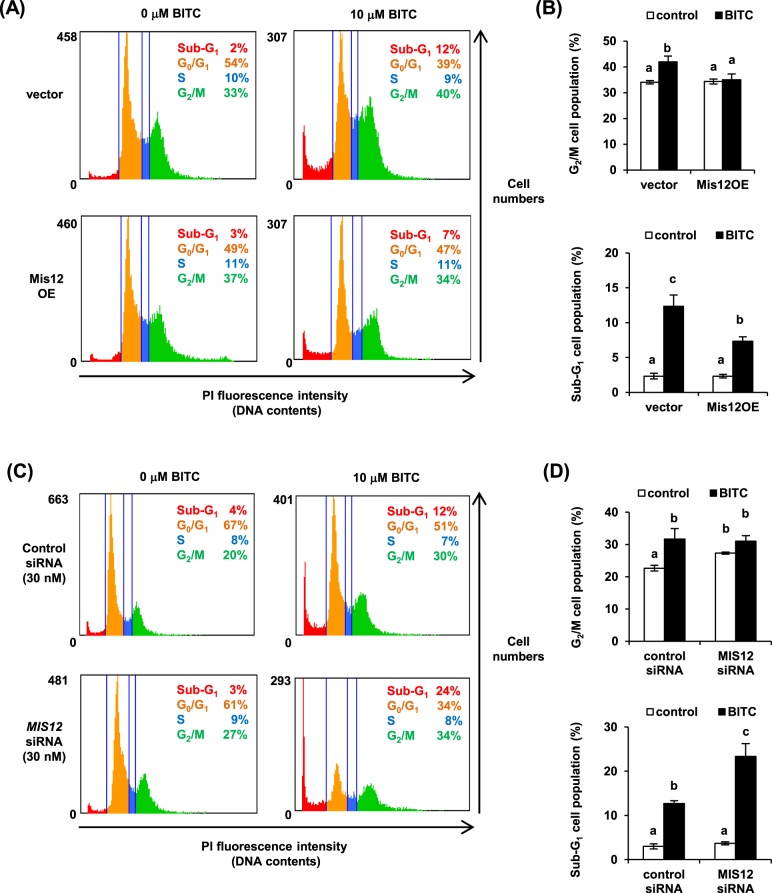
Change in Mis12 protein level affects the sensitivity of cells to BITC-induced apoptosis. (**A**,**B**) Cells in the Mis12 OE and vector group were treated with BITC for 24 h and subjected to a cell cycle distribution analysis using Tali™ image-based cytometer. The representative histogram is shown in (**A**). The G_2_/M and Sub-G_1_ cell populations were statistically analyzed in (**B**). (**C**,**D**) HCT-116 cells were transfected with the control siRNA or Mis12-specific siRNA. After the treatment with BITC for 24 h, the cell cycle distribution was analyzed as (**A**). The representative histogram is shown in (**C**). The G_2_/M and Sub-G_1_ cell populations were statistically analyzed in (**D**). The values represent means ± SEM of three separate experiments. Data were analyzed by a one-way analysis of variance (ANOVA) followed by multiple comparisons among the means (Tukey’s HSD) using XLSTAT software (Addinsoft, Paris, France). Different letters above the bars indicate significant differences among the treatments for each compound (*P* < 0.05).

The BITC-induced chromosome misalignment may cause delay in mitosis. Consistently, BITC delays the cell cycle progression at G_2_/M phase and induces G_2_/M phase-specific apoptotic cell death in human leukemia Jurkat cells. Thus, we hypothesized that the decrease in Mis12 enhances the BITC-induced apoptosis by increasing the G_2_/M cell population in HCT-116 cells. To test this hypothesis, we examined the effect of the overexpression or knockdown of Mis12 on cell cycle regulation by BITC. In the vector group, BITC increased the G_2_/M and Sub-G_1_ cell population (Fig. 6A,B), the latter of which is characterized as the apoptotic cells^26^. On the other hand, the Mis12 overexpression cancelled the BITC-increased G_2_/M cell population and partly, but significantly, attenuated the BITC-increased Sub-G_1_ cell population. Compared with the control siRNA-transfected group, Mis12 knockdown alone increased the G_2_/M cell population, but not the sub-G_1_ cell population (Fig. 6C,D). Moreover, the increase in the sub-G_1_ cell population by BITC was enhanced by the Mis12 knockdown. These data suggested that BITC increases the G_2_/M cell population, possibly by reducing the Mis12 level and thus sensitizing the cells to the BITC-induced apoptosis.

## Discussion

In the present study, we originally developed the yeast screening system as a reliable and convenient tool to identify overexpression suppressors for the antiproliferative effect of BITC. As shown in Table 1, the 12 identified genes in yeast includes ATP synthase (*ATP10*), amino acid transport system (*BAP2* and *STP1*), cytochrome c oxidase Cu chaperon (*COX23*), kinetochore complex (*MTW1*), deubiquitinating enzymes (DUBs) (*UBP7*), ribosomal RNA (rRNA) methyltransferase (*RCM1*) and protein phosphatase type 2A (PP2A) complex (*RTS3*). According to the *Saccharomyces* genome database, Bap2, Cox23, Mtw1, Ubp7 and Rcm1 are recognized as the human homologs of SLC7A5-10, CHCHD7, Mis12, USP2, 8, 21 and NSUN5, respectively. Because the structure is highly similar between homologs and the overexpression of each gene increases the resistance against BITC, they might play a pivotal role in the BITC-induced antiproliferation in both the yeast and human cells. Among the identified 12 factors, ATP synthase, cytochrome c oxidase Cu chaperon and DUB have already been identified as the direct binding targets for PEITC, an aromatic ITC analogue of BITC, by an *in vitro* proteomics study using the human cancer cell lysate^13^. The existence of such common hits between our screening and previous proteomics analysis further confirmed the usefulness of our yeast screening system and also implies that the down-regulation or dysfunction of these proteins by the direct binding of BITC contributes to its antiproliferative effect. For example, the collapse of the mitochondrial electron transport system through the direct binding of BITC to ATP synthase or cytochrome c oxidase Cu chaperon might induce apoptosis. This idea is supported by the finding that the mitochondrial death pathway is involved in BITC-induced apoptosis^7^.

Our screening system identified other types of factors contributing to the BITC resistance such as the components of the amino acid transport system (Bap2 and Stp1). Bap2 is the branched-amino acid transporter that mediates the uptake of leucine, isoleucine and valine to the cells^27^. Stp1 regulates the transcription of amino acid transporters including Bap2^28,29^. The increased expression of the L-type amino acid transporters (LATs), the major transporters that regulate uptake of leucine to cells, is the hallmark of many kinds of human cancers^30^. There is now strong evidence that leucine enhances the mammalian target of the rapamycin complex 1 (mTORC1) signaling which modulates protein synthesis to regulate cell growth, proliferation and cellular differentiation^31^. Furthermore, we have recently reported that an mTOR inhibitor, NVP-BEZ235, potentiated the BITC-induced apoptosis induction in human colorectal cancer cells^32^. Thus, the inhibition of the mTORC1 pathway through the down-regulation of the LATs might contribute to the antiproliferative effect of BITC in human cells. Similarly, the attenuation of the antiproliferative effect of BITC by overexpressing the rRNA methyltransferase might be attributed to the facilitation of protein synthesis because methylation of the rRNA regulates ribosome maturation^33^.

In this study, we identified a kinetochore protein Mtw1, a well-conserved orthologue of human Mis12, as one of the overexpression suppressors for the antiproliferative effect of BITC using the yeast screening system. Our data showed that the antiproliferative effect of BITC was not only inhibited by the Mis12 overexpression in HCT-116 cells, but also enhanced by the Mis12 knockdown (Fig. 4C,F). The Mis12 knockdown alone induced G_2_/M delay but not apoptosis in HCT-116 cells and synergistically enhanced the antiproliferative effect of BITC accompanied with acceleration of the BITC-induced apoptosis (Figs 4F and 6). These observations suggested that the accumulation of the G_2_/M cell population is involved in the increased sensitivity to the BITC-induced apoptosis. This idea is supported by the report showing that BITC induces the G_2_/M phase-specific apoptosis in human leukemia Jurkat cells^5^. PP2A, a factor identified by our screening, is a family of serine/threonine protein phosphatases that regulates many cellular processes including cell cycle regulation, apoptosis, protein synthesis, cell morphology and development^34,35^. Down-regulation of the PP2A-B55 complex accelerates entry into mitosis and inhibits exit from mitosis. The inactivation of PP2A induces apoptosis in many cancer cells^36^. Based on these findings, the overexpression of the PP2A component might weaken the antiproliferative effect of BITC by inhibiting the BITC-induced M delay and apoptosis. However, PP2A regulates cell growth both positively and negatively due to the formation of a multitude of different PP2A holoenzyme complexes^37^. Thus, the individual role in each of the PP2A complexes in the antiproliferative effect of BITC needs to be clarified.

We also found that BITC enhanced Mis12 phosphorylation and tended to induce Mis12 ubiquitination (Fig. 5I) and decreased Mis12 in a proteasome-dependent manner (Fig. 5H), suggesting that the post-transcriptional regulation of Mis12 plays a key role in the antiproliferative effect of BITC. Although previous research indicated that Mis12 is phosphorylated at Ser190 or Thr192, and Ser213 in *Schizosaccharomyces pombe* possibly by Gsk3 kinase^24^, the mechanism stabilizing kinetochore attachment of Mis12 is still unknown. The phosphorylation sites of human Mis12 and their effect on the stability should be elucidated in the future researches. We identified *UBP7* encoding DUB, a large group of proteases that cleave ubiquitin from proteins and other molecules including ubiquitin-specific proteases (USPs) and ubiquitin C-terminal hydrolases (UCHs). Since dysregulation of the ubiquitin-proteasome system has been suggested to play an important role in the pathogenesis of many human diseases including cancer^38^, DUBs are promising targets for cancer treatment through the interference with the ubiquitin regulation machinery^39^. For example, the knockdown of USP7 actually exerts an antiproliferative effect through induction of cell cycle arrest in HCT-116 cells^40^. Since proteomics analysis also identified DUB as a direct target of the ITC compound^13^, the DUB dysfunction through the direct binding of BITC might contribute to the Mis12 degradation by the ubiquitin-proteasome system. In fact, a very recent study revealed that BITC and PEITC inhibit the activity of USP9x and UCH37, possibly through the direct modification to the Cys residue in the catalytic domain of the DUBs^41^, supporting our hypothesis. However, there is another possibility that the phosphorylation and ubiquitination of Mis12 is followed by the BITC-induced cell cycle delay. Therefore, the further studies on the upstream signaling of Mis12 phosphorylation are required. Since the ITCs-bound proteins are also degraded in a proteasome-dependent manner, the direct modification of the target proteins by BITC is another possible mechanism of the Mis12 down-regulation. A recent study revealed that the subunits of the multi-protein complex including the MIND kinetochore complex composed of Mtw1, Dsn1, Nsl1 and Nnf1 were subjected to tuning toward the fine proportion at the protein level by the ubiquitin-proteasome system in yeast^42^. We preliminary found that BITC drastically decreased the protein level of the human Dsnl in HCT-116 cells (data not shown). These results suggest that Mis12 affects the stability of the subunits of the human Mis12 complex other than Mis12 and becomes the promising target of the anti-cancer agent.

The human Mis12 kinetochore complex, a heterotetramer composed of Mis12, Dsn1, Nsl1and Nnf1, connects the centromere region on the chromosome to the mitotic spindle microtubule and is essential for the kinetochore assembly and proper chromosome segregation^24,25^. Mis12 depletion induces the high frequency of misaligned metaphase chromosomes and lagging chromosomes in anaphase, followed by the frequent appearance of micronuclei in the interphase. These mitotic and interphase abnormalities should cause chromosome missegregation and aneuploidy, an aberrant number of chromosomes^24,43^. Consistent with these reports, we observed chromosome misalignment induced by BITC (Fig. 5F). This result indicated that BITC induces aneuploidy through the down-regulation of Mis12. Targeting the components involved in the mitotic regulation is a major strategy of cancer therapy, whereas the induction of delay in mitosis causes not only the cell death but also aneuploidy^44^. Recent study revealed that paclitaxel, a first-line anti-cancer agent inhibiting the depolymerization of spindle microtubules, induces aneuploidy to suppress proliferation in a clinically relevant concentration range in breast cancer^45^. Paclitaxel as well as BITC decreased the protein level of Mis12 at the concentration where it suppresses cell proliferation in HCT-116 cells (Fig. 5J,K). These findings suggest that the induction of aneuploidy is an effective strategy for cancer therapy, while aneuploidy has ever been recognized to be a hallmark of cancer development. Some researchers argue that aneuploidy is simply a by-product of tumorigenesis^46^, while others argue that aneuploidy is the driving force of tumorigenesis^47^. This confusion may be due to various factors including the heterogenic nature of cancers and the individual genetic alteration in humans^48^. Since BITC decreased the protein level of Mis12 in non-tumorigenic HaCaT cells as well as in tumorigenic HCT-116 cells (Fig. 5B,C), it is very important to examine whether or not the down-regulation of Mis12 by BITC causes carcinogenesis in various cell types including normal cells. Although the agents targeting the components of kinetochore complex including Ndc80 and Nuf2 have been shown to have anti-cancer effects^49–51^, this is the first report, to the best of our knowledge, to show the mechanism to suppress cancer cell proliferation through the degradation of Mis12. This finding is expected to contribute to the development of the new anti-cancer drug targeting Mis12 as well as other kinetochore complex members.

**Figure 7:**
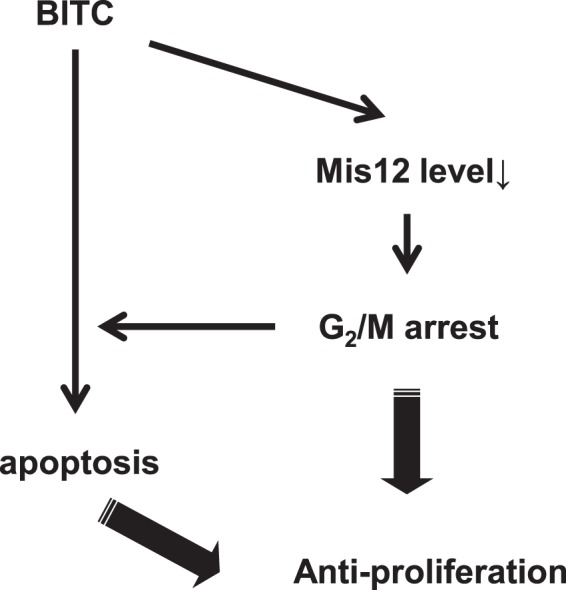
Proposed model of antiproliferation by BITC. BITC has been shown to induce G_2_/M arrest and apoptosis in various cancer cells. BITC possibly decreases Mis12 protein level by inducing the phosphorylation, ubiquitination and proteasomal degradation of it. The reduced-Mis12 level causes G_2_/M delay, but not apoptosis and enhances the apoptosis induction by BITC. Thus, the down-regulation of Mis12 contributes to the antiproliferative effect of BITC by inducing G_2_/M delay and sensitizing cells to the BITC-induced apoptosis.

Here, we proposed the regulating role of Mis12 in the BITC-induced suppression of proliferation in HCT-116 cells due to G_2_/M delay and apoptosis in Fig. 7. However, the other study implies that the cell cycle inhibition by ITCs is caused through the disruption of spindle structure by the ITC binding to tubulin^10^. Furthermore, the decrease of Mis12 level by paclitaxel, which is known to cause the spindle disruption up to the dose, suggests the probability that the spindle disruption leads to the downregulation of Mis12. Thus, it is also possible that the disruption of spindle structure by BITC is followed by cell cycle inhibition and Mis12 degradation. Even though the Mis12 overexpression canceled the cell cycle inhibition by BITC (Fig. 6B), the excess Mis12 may impair the disruption of spindle structure to interfere with the BITC binding to tubulin. Future studies should be directed to clarify the detailed mechanism of the down-regulation of Mis12 level by BITC. Our yeast screening system requires a lower cost, shorter time and simpler operations compared to the human cell screening system. We also revealed the commonality between the molecules identified by our screening and possible binding targets of BITC previously reported^13^, suggesting the high utility of our screening system. Although we did not provide evidence that BITC targets Mis12 directly or indirectly, future efforts will be concerned with chemical detection of the BITC-modified Mis12 by mass spectrometry or by immunoprecipitation using the antibody recognizing the Lys adduct of BITC^52^. Furthermore, because inhibition of the DUB activity by BITC is the most plausible indirect pathway, it should be clarified whether the DUB modification by BITC contributes to the proteasome-dependent Mis12 degradation.

## Materials and Methods

### Chemicals and antibodies

BITC was purchased from LKT Laboratories, Inc. (St Paul, MN). Antibodies against Mis12 (#98368, 1:200), ubiquitin (#sc-8017, 1:200) and actin (#sc-8432, 1:200), and horseradish peroxidase-linked anti-rabbit and anti-mouse IgGs, Mis12-specific siRNA, control siRNA, siRNA transfection medium, siRNA transfection reagent and Protein A/G PLUS-Agarose Immunoprecipitation reagents were purchased from Santa Cruz Biotechnology (Santa Cruz, CA). Antibody against phosphoserine/threonine/tyrosine (#ab15556, 1:1000) was purchased from abcam (Cambridge, United Kingdom). Peroxidase anti-peroxidase soluble complex (PAP) antibody (#P3039, 1:1000), propidium iodide (PI) and RNase A were purchased from Sigma-Aldrich (St. Louis, MO). Antibody against HA-Tag (#3724, 1:1000) was purchased from Cell Signaling Technology, Inc. (Beverly, MA). Alexa Fluor-linked anti-rabbit and anti-mouse IgGs, McCoy’s 5A, penicillin/streptomycin, trypan blue stain, FastDigest EcoRI, FastDigest HindIII, Pierce^®^ BCA Protein Assay Kit and Trizol^®^ reagent were purchased from Thermo Fisher Scientific (Waltham, MA). Fatal bovine serum (FBS) was purchased from Nichirei Corporation (Tokyo, Japan). Immobilon-P membrane was purchased from Merck Millipore (Billerica, MA). Taq polymerase and PrimeScript^TM^ RT Master Mix (Perfect Real Time) were purchased from Takara Bio Inc. (Shiga, Japan). Paclitaxel was purchased from Wako Pure Chemical Industries (Osaka, Japan). All other chemicals were purchased from Nakalai Tesque Inc. (Kyoto, Japan).

### Yeast strain, growth conditions and yeast transformation

*S. cerevisiae* strain BY4741 (*MATa his3Δ1 leu2Δ0 met15Δ0 ura3Δ0*)^53^ was used for yeast screening system. *S. cerevisiae* MTW1-TAP strain obtained from Funakoshi Co., Ltd. (Tokyo, Japan) was used for Western blot analysis. Yeast cultivation and transformation were performed as previously described^54^. SC-His was used for the selection of yeast transformed with plasmid.

### Generation of gene overexpression library of yeast

To generate the gene overexpression library of *S. cerevisiae*, BY4741 strain was transformed with pRS423ks and plated on minimal media lacking His. Because BY4741 strain harbors inactivated *his3* genes, it cannot grow on His-lacking medium. However, the genomic DNA of the yeast strain from which the library was constructed has the functional *HIS3* genes. Thus, the presence of a plasmid in the library that contains *HIS3* genes in BY4741 strain will restore growth to a transformant on medium lacking His.

### Measurement of maximum growth rate

Yeast growth was measured by monitoring OD595 every 30 min at 30 °C using a microplate reader (Model 680XR, Bio-Rad Laboratories, Hercules, CA). Maximum growth rate was calculated as described previously^54^.

### Screening for overexpression suppressors of antiproliferation by BITC in yeast

The entire scheme of yeast screening system is described as follows. A genome-wide multi-copy plasmid library of *S. cerevisiae* cloned into pRS423ks^22^ was divided to 8 groups for convenience and introduced into BY4741 cells. The 8 groups of transformants were cultivated in SC-His medium until mid-log phase, seeded to 96 well-plate and cultured in YPD liquid medium with 100 μM BITC for 3 days at 30 °C. The cell suspension in each well was plated onto SC-His agar plate and incubated for 3 days at 30 °C. Relatively big colonies were picked up and subjected to colony PCR using primers 5′-AAGATCAACACTGCCAAAGCG-3′ and 5′-AGCCATATCAATACGGCGAACATC-3′ to amplify DNA sequence of gene inserted in plasmid. PCR product was digested with EcoRI and HindIII for 1 h at 37 °C and separated by 1% agarose gel electrophoresis. Plasmid DNA from colony with the same band pattern frequently seen here (more than 2 times) was isolated by Yeast DNA miniprep as previously described^54^. *Escherichia coli* XL1-Blue Electroporation Competent Cell (Agilent Technologies, Santa Clara, CA) was transformed with the isolated yeast DNA by electroporation and plated on ampicillin-containing LB plate. Plasmid DNA was isolated by *E. coli* DNA miniprep as previously described^54^. DNA sequence of insert in the plasmid was determined by the service of Macrogen japan (Tokyo, Japan) using primers 5′-CGGCCGCTCTAGAACTAGTGGATCC-3′ and 5′-ATTGGGTACCGGGCCCCCCCTCGAG-3′. To re-evaluate the suppressor activity of the identified candidate gene, suppressor plasmid was transfected to yeast again and performed spot assay.

### Spot assay

Yeast cells were cultured in YPD liquid medium with BITC (100, 500 and 1000 µM) or 0.1% DMSO for 30 min. The cell suspension was diluted 1, 100 and 500 times with YPD liquid medium and the diluent of cell suspension (10 μl) was spotted onto YPD agar plate. After 2 days cultivation, the number of colonies was counted.

### Human cell line and cell culture

Human colon cancer cell line HCT-116 was obtained from the American Type Culture collection (ATCC) (Manassas, VA). HCT-116 cells were maintained in McCoy’s 5A medium supplemented with 10% heat-inactivated FBS and 1% penicillin/streptomycin. Immortalized non-tumorigenic human epidermal cell line HaCaT was kindly provided by Shiseido Research Center (Yokohama, Japan). HaCaT cells were maintained in Dulbecco’s modified Eagle’s medium supplemented with 10% heat-inactivated FBS and 0.5% penicillin/streptomycin. Cells were grown at 37 °C in an atmosphere of 95% and 5% CO_2_.

### Western blot analysis

The experiments for yeast cells^55^ and for human cells^56^ were performed as previously described. Briefly, whole cell lysates were subjected to SDS-PAGE and transferred to PVDF membrane and the chemiluminescent picture was taken after immunoblotting using antibodies. Densitometric analysis of the bands was carried out using the Image J Software Program.

### RNA interference

Cells were cultured in 6-well plates (2 × 10^5^ cells per well) in normal growth medium without antibiotic and transfected with siRNA. Predesigned siRNA targeting Mis12 or nonspecific control siRNA were transfected to the cells according to the manufacturer’s instructions using siRNA transfection medium and siRNA transfection reagent. After the incubation for appropriate time, cells were assayed using the appropriate protocol.

### Trypan blue dye exclusion assay

Trypan blue dye exclusion assay was carried out for quantitative analysis of cell viability. Cell suspensions were mixed with 0.4% Trypan blue stain. Viable cells (cells that excluded blue dye) were counted using a hemocytometer (Bürker-Türk) under a light microscope.

### MTT assay

MTT assay was carried out for quantitative analysis of cell viability as previously described^56^. Briefly, cells were treated with MTT solution and the absorbance was measured at 570 nm after 2 h-incubation at 37 °C.

### RT-PCR

Cells were washed with ice-cold phosphate buffered saline (PBS) (−). Total cellular RNA was isolated using Trizol reagent according to the manufacturer’s recommendations. RNA was quantified by measuring absorbance at 260 nm. Total RNA (0.5 μg) was reverse transcribed using PrimeScript^TM^ RT Master Mix (Perfect Real Time). PCR amplification was then performed with Taq polymerase and specific primers. Primers used in PCR amplification are as follows: *MIS12*, 5′-CAGGCCGTTGAACAGGTTAT-3′ and 5′-TCAGCTGCAAAAACAGTTGC-3′ (160 bp); and *β-ACTIN*, 5′-GTCACCCACACTGTGCCCATCTA-3′ and 5′-GCAATGCCAGGGTACATGGTGGT-3′ (455 bp). The PCR products were then subjected to agarose gel electrophoresis (3%), stained with ethidium bromide and photographed. Densitometric analysis of the bands was carried out using the Image J Software Program.

### Plasmid and stable cell line

Plasmid to express Mis12 was kindly provided by Drs. Mitsuhiro Yanagida and Takeshi Hayashi (Okinawa Institute of Science and Technology Graduate University, Okinawa, Japan). Mis12 cDNA fused with HA-tag sequence at N-terminal was produced by PCR and subcloned into pQCXIP (Clontech, Mountain View, CA). HCT-116 cells stably expressing HA-Mis12 were established as previously described^57^.

### Immunoprecipitation assay

Cells were washed with ice-cold PBS (−), lysed in RIPA buffer (50 mM Tris-HCl pH 7.4, 150 mM NaCl, 1% NP-40, 0.25% Sodium deoxycholate and 1 mM EDTA) containing protease inhibitor cocktail and phosphatase inhibitor cocktail, and left on ice for 15 min. After sonication, lysates were centrifuged and the supernatant was used as whole cell lysates. Whole cell lysates (800 μg protein) were incubated with HA antibody for 24 h at 4 °C. Then, 20 μl Protein A/G PLUS-Agarose Immunoprecipitation reagents were added and the mixture was incubated for 2 h at 4 °C. Beads were washed with lysis buffer three times. Immunoprecipitated proteins were subjected to Western blot analysis.

### Cell cycle analysis

After treatment, the culture supernatant was collected, and the cells were detached by trypsin treatment. The detached cells were suspended in the collected culture supernatant. After the centrifugation at 1,500 rpm for 5 min at 4 °C, cells were stained with PI solution (20 μg/ml PI, 0.1% Triton X-100, 0.2 μg/ml RNase A in PBS (−)) for 30 min under dark condition at room temperature. Stained cells were analyzed by a Tali™ Image Based Cytometer (Thermo Fisher Scientific, Waltham, MA).

### Immunofluorescence microscopy

Cells on coverslips were fixed with 4% paraformaldehyde (PFA) in PBS for 15 min, permeabilized with 0.1% Triton X-100 in PBS for 5 min at room temperature. The cells were blocked with 4% bovine serum albumin (BSA) in PBS for 15 min, and then incubated for 15 min with primary antibodies diluted in 4% BSA in PBS. The cells were washed three times with PBS, and incubated for 15 min with secondary antibodies conjugated to Alexa fluorophors. After washing the cells, the coverslips were mounted on microscope slides and imaged using a IX71 fluorescent microscope equipped with a 60 × objectives (Olympus, Tokyo, Japan). Image data were processed and quantified using Image J software. The signal intensity of HA-Mis12 was determined by measuring the integrated fluorescence intensity within a 3 × 3 pixel square positioned over a single HA-Mis12 signal and subtracting the background intensity of a 3 × 3 pixel square positioned in a corresponding background. A minimum of 3 cells and 10 kinetochore foci per cell were measured for each condition each experiment (each replicated a minimum of three times).
